# Crucial role of zebrafish *prox1 *in hypothalamic catecholaminergic neurons development

**DOI:** 10.1186/1471-213X-8-27

**Published:** 2008-03-10

**Authors:** Anna Pistocchi, Germano Gaudenzi, Silvia Carra, Erica Bresciani, Luca Del Giacco, Franco Cotelli

**Affiliations:** 1 Department of Biology, Università degli Studi di Milano, Via Celoria 26, 20133 Milano, Italy

## Abstract

**Background:**

*Prox1*, the vertebrate homolog of *prospero *in *Drosophila melanogaster*, is a divergent homeogene that regulates cell proliferation, fate determination and differentiation during vertebrate embryonic development.

**Results:**

Here we report that, in zebrafish, *prox1 *is widely expressed in several districts of the Central Nervous System (CNS). Specifically, we evidenced *prox1 *expression in a group of neurons, already positive for *otp1*, located in the hypothalamus at the level of the posterior tuberculum (PT). Prox1 knock-down determines the severe loss of hypothalamic catecholaminergic (CA) neurons, identified by tyrosine hydroxylase (TH) expression, and the synergistic *prox1/otp1 *overexpression induces the appearance of hypothalamic supernumerary TH-positive neurons and ectopic TH-positive cells on the yolk epitelium.

**Conclusion:**

Our findings indicate that *prox1 *activity is crucial for the proper development of the *otp1*-positive hypothalamic neuronal precursors to their terminal CA phenotype.

## Background

The catecholaminergic neurons of the CNS of vertebrates participate in a wide variety of tasks, including motor coordination, mood regulation, and cognitive function, among others. Neurotransmitters catecholamines (CA), namely Dopamine (DA), Adrenaline (AD), and Noradrenaline (NA), are neuroactive molecules that exert strong influence on vertebrates behavior [1] and serve a variety of central and peripheral functions [2].

Embryological studies indicate that several extracellular signals, as Hedgehog and FGF, are vital to define the development of the prosencephalic CA neurons [3-7]. The homeodomain transcription factor Orthopedia (Otp), regulated by such signaling pathways [8], is crucial in restricting the fate of multiple classes of secreting neurons in the neuroendocrine hypothalamus of vertebrates [9,10]. Specifically, Otp is required for the correct differentiation of the CA neurons positioned in the zebrafish Posterior Tuberculum (PT) and hypothalamus [8,11]. Despite all these evidences, the role of specific transcription factors leading to the proper differentiation of the hypothalamic CA neurons remains largely unclear [7].

*Prox1 *homeobox gene is the vertebrate homologous of *prospero *in *Drosophila melanogaster*. During *Drosophila *embryonic development, *prospero *is expressed in neuronal precursors and determines the neuronal/glial fate of sibling cells [12,13]. *prospero/Prox1*'s high level of homology pinpoints possible functional conservation through evolution, suggesting *Prox1 *involvement in vertebrate cell fate determination. Indeed, also during murine brain development, *Prox1 *is expressed in most of the locations in which neurogenesis and glial formation occur during middle and late prenatal and postnatal stages, as the subventricular zone, several regions of the prethalamus and hypothalamus, the cerebellum, and the hippocampus [14].

Here, we demonstrated that, in zebrafish, *prox1 *is widely expressed in the developing CNS, and one of its expression domains is located in the area corresponding to the ventral part of the PT and the adjacent hypothalamic district, the area hosting the cluster of CA neurons positive for *otp1 *expression [8,11]. Moreover, we took advantage of the zebrafish animal model to investigate the *in vivo *influence of *prox1 *on hypothalamic CA neuronal development by means of morpholino- and mRNA- loss and gain of function methodologies.

We provide evidence that *prox1 *is required for the development of hypothalamic neuronal progenitors into mature CA neurons.

## Results and discussion

Homeobox genes are expressed in a temporal and spatial restricted manner and play crucial roles for cell type specification [15,16]. Zebrafish *prox1 *is a divergent homeodomain transcription factor whose homologues in *Drosophila *and mice regulate cell proliferation, fate determination and differentiation in CNS and sensory tissues [17-21]. Noteworthingly, during murine brain development, *Prox1 *is also expressed in the hypothalamus [14], where several CA neurons differentiate.

### Spatio-temporal expression of prox1 during embryogenesis

**Figure 1:**
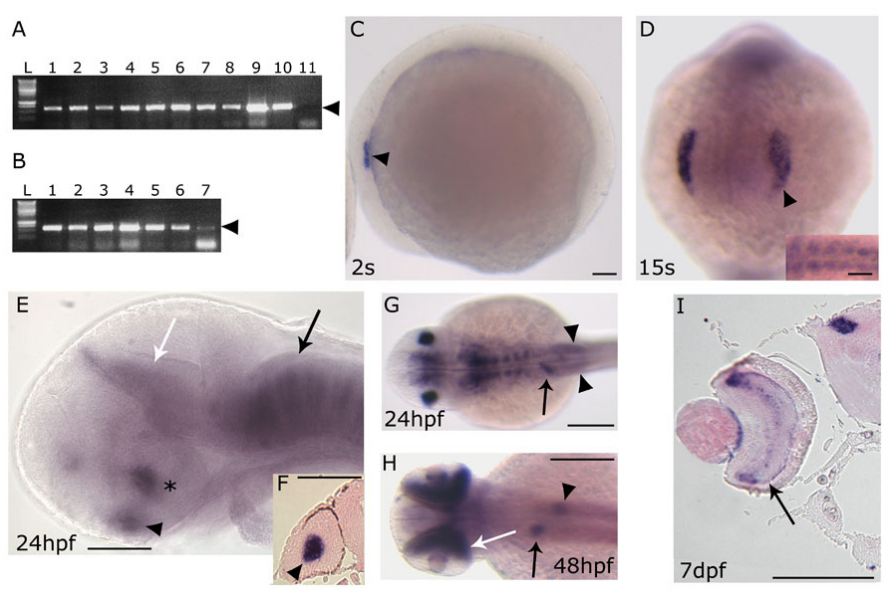
***prox1 *temporal and spatial expression pattern analyzed by RT-PCR and *in situ *hybridization**. *(A) *RT-PCR performed on different embryonic stages: 1–2 cells stage (lane 1), 30% epiboly (lane 2), 50% epiboly (lane 3), 80% epiboly (lane 4), tail bud (lane 5), 8 somites (lane 6), 15 somites (lane 7), 24 hpf (lane 8), 72 hpf (lane 9), 5 dpf (lane 10) and negative control (lane 11) in the absence of cDNA. *(B*) RT-PCR performed on different adult organs: DNA ladder (L), testis (lane 1), overy (lane 2), gills (lane 3), gut (lane 4), eye (lane 5), brain (lane 6) and liver (lane 7). Arrowhead indicates the size of the *prox1*-specific PCR product (620 bp). *(C-I) prox1 *WISH *(C) *the first signals appeared at 2 s in the otic placode (arrowhead). *(D) *at 15 s the signal is detected in the lens placode (arrowhead), and somites (inset). *(E) *at 24 hpf *prox1 *is expressed the hypothalamus (asterisc), the pituitary (black arrowhead), the pretectal segment (prosomere 1) (white arrow), as well as segmentally arranged cells of the hindbrain (black arrow). *(F) *transverse section through the forebrain of a 24 hpf stage zebrafish embryo shows the signal in the lens (black arrowhead). *(G) *at 24 hpf additional *prox1 *signals are present in the liver primordium (arrow), and posterior lateral line primordium (arrowheads). *(H) *later during development, (48 hpf) *prox1 *expression is detected in distinct domains in the liver (arrow) and pancreas (arrowhead), while a further signal appeares in the retina (white arrow). *(I) *transverse section through the forebrain of a 7 dpf stage zebrafish larva shows the signals in the retina inner nuclear layer (arrow) and in the pretectal nuclei (arrowhead). *(C,E) *Lateral views are shown. *(D) *Frontal view is shown. *(G,H) *Dorsal views are shown. Anterior is always to the left. Scale bars indicate 100 μm (*A,B,C,D,G,H,I*) or 200 μm (*E,F*).

Previously published immunostaining analysis of the Prox1 expression pattern revealed that the gene is active in several zebrafish embryonic districts [22]. To explore the role of *prox1 *during zebrafish CNS development, we first performed a more detailed characterization of *prox1 *expression during embryogenesis and in adult organs by means of RT-PCR (Fig. 1). We detected the presence of *prox1 *transcript at all stages analyzed, including the zygote, indicating that *prox1 *is also maternally expressed (Fig. 1A). Furthermore, we report *prox1 *expression in the adult brain, eyes, and in non-neuroectodermal territories (testis, ovary, gills, gut, liver) (Fig. 1B). The spatial and temporal distribution of *prox1 *transcripts was then examined by whole mount *in situ *hybridization (WISH), following standard protocols, with digoxigenin- and fluorescein-UTP-labeled probes [23]. At all stages analyzed, from 1–2 cell stage to 5 days post fertilization (dpf), zebrafish *prox1 *expression analysis confirms and improves previous immunostaining results [22]. From an evolutionary point of view, the striking resemblance of the *prox1 *expression pattern among vertebrates strongly suggests a conserved role for the gene during evolution. Although RT-PCR revealed the presence of maternal and zygotic transcripts, *prox1 *mRNA is first detectable through WISH around the 2 somites (s) stage in the ectodermic region corresponding to the otic placode (Fig. 1C). At 15 s stage, *prox1 *is also expressed in the lens placode (Fig. 1D) and in the first formed somites (Fig. 1D, inset). At 24 hours post fertilization (hpf), *prox1 *expression persists in the lens (Fig. 1E,F) and in the adaxial cells (see Additional file 1) that will later differentiate in slow muscle fibers [24]. Moreover, two distinct bilateral *prox1 *signals appear on each side of the midline, at the rostral end of the neural tube, in a region corresponding to the hypothalamus (Fig. 1E). At the same stage, strong *prox1 *expression signals define the pituitary, the pretectal segment (prosomere 1, according to Rink and Wullimann [25]), and each hindbrain neuromeric segment (rhombomeres), where *prox1 *expression is visible in segmentally arranged clusters of cells (Fig. 1E). Additionally, *prox1 *signals are detectable in the liver and the posterior lateral line primordium (PLLP) (Fig. 1G). Starting from 48 hpf, further signals appear in the retina, pancreas (Fig. 1H), and in the cephalic ganglia (see Additional file 2). At 7 dpf, when the retinal's layers are fully differentiated, *prox1 *signal is detectable specifically in the inner nuclear layer, as previously shown in other vertebrates [20], and in the pretectal nuclei (Fig. 1I). In this work, we focused our attention on *prox1 *role during zebrafish hypothalamic development.

### prox1 is required for the development of a group of hypothalamic CA neurons

The determination of the neurotransmitter phenotype is an important aspect of neuronal differentiation. Degeneration of substantia nigra DA neurons in humans is a hallmark of Parkinson's disease, and the malfunction of CA neurons in other brain regions is implicated in psychiatric disorders and neuroendocrine dysregulation [26-28].

**Figure 2:**
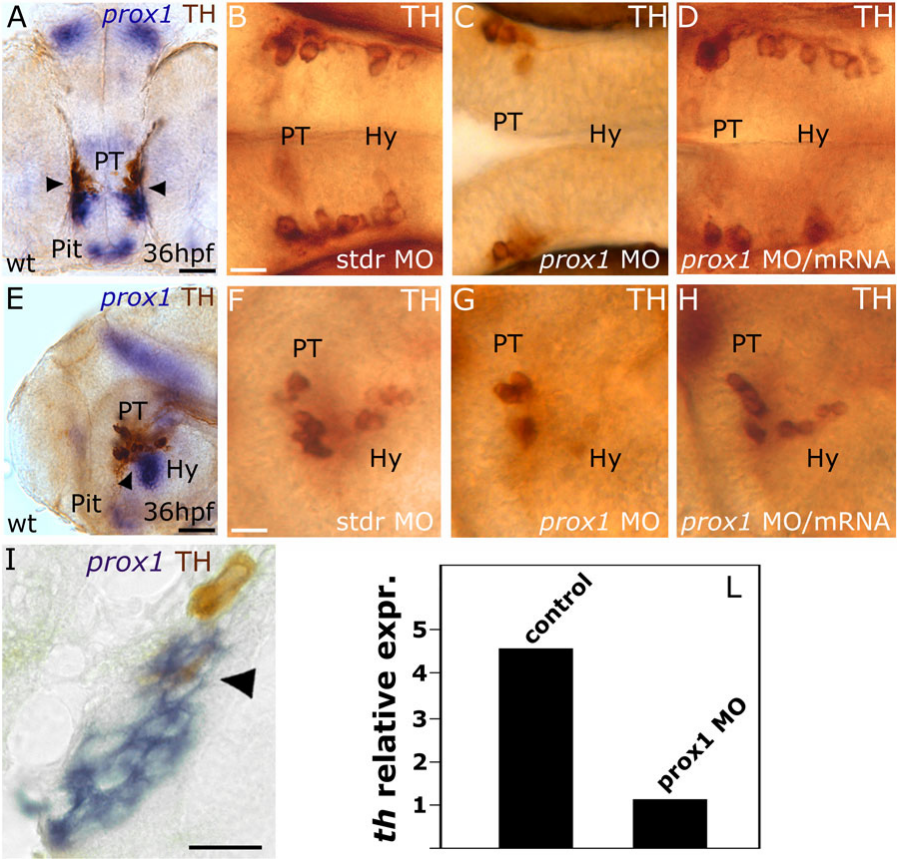
***prox1 *is required for the development of CA neurons in the hypothalamus**. Anterior is left in all panels except *(A)*, frontal view. Dorsal is up, except for (*B,C,D*), dorsal view. Eyes or lens have been removed for better lateral viewing. (*A,E,I*) *prox1 *WISH combined with TH immunohistochemistry. Anti-TH antibody labels the PT and hypothalamic CA neurons at 36 hpf. Colabelling with *prox1 *is evident in a fraction of TH-positive neuroblasts in the hypothalamus (arrowheads), as also confirmed by the longitudinal section of the embryo (*I*). *(C,G) *microinjection of *prox1 *MO lowers the number of TH-labelled CA neurons in the hypothalamus in comparison to standard control injected embryos *(B,F)*. *(D,H) *coinjection of *prox1 *mRNA and *prox1 *MO rescued the morphant phenotype. (*L*) Quantitative real time RT-PCR. TH-specific mRNA is almost five-fold decreased following *prox1 *MO injection. The result represents at least three independent experiments, and 18S was used as an internal control. The following abbreviations are used: posterior tuberculum (PT), pituitary (Pit), hypothalamus (Hy), standard control morpholino oligonucleotide (stdr MO). Scale bars indicate 10 μm *(B,F,I) *or 20 μm *(A,E)*.

In zebrafish, a detailed characterization of the CA neurotransmitter pathway makes this organism a favorite model to address the ontogeny of the vertebrate neurosecretory system [8,11,29-31]. TH-expressing CA neurons are primarily located in the anterior dorsal telencephalon and hypothalamus of the developing forebrain, with a few additional neurons present near the postoptic commissure and pretectum region [29]. 36 hpf embryos hybridized with *prox1 *mRNA probe and immunostained with TH antibody show that *prox1 *transcript is present in close proximity to the most caudal posterior tubercular and the adjacent hypothalamic TH-expressing cells, with partial overlap of the two signals (Fig. 2A,E,I). To determine whether *prox1 *is required for CA neuron development, we knocked-down the protein level by injecting 4 ng/embryo of a specific ATG-targeted morpholino oligonucleotide (*prox1 *MO) [32,33]. Abrogation of Prox1 function leads to a severe loss of neurons in the hypothalamic portion of the PT/hypothalamic CA cluster (at 36 hpf, 70% of the embryos showing no or few TH-positive cells in this area, n = 150) (Fig. 2B,C,F,G). This defect is already evident at 24 hpf (see Additional file 3) and persists later during development, as shown by TH immunostaining at 48 hpf (see Additional file 3). The overall architecture of the ventral diencephalon was not affected in *prox1 *MO injected embryos, as suggested by the normal expression of *shh *(see Additional file 4). Therefore, we concluded that the decrease in the number of CA cells is not determined by an alteration in the patterning of the hypothalamus. We also analyzed the *th *expression levels in *prox1 *MO injected embryos by means of quantitative real time RT-PCR. The *th *specific mRNA level was about five-fold decreased in *prox1 *MO injected embryos when compared to the *th *expression in control embryos injected with the standard control morpholino oligonucleotide (stdr MO) (Fig. 2L), confirming the immunohistochemical analysis reported above. To demonstrate that the reduction of TH positive cells in the hypothalamus of the embryos is specifically caused by the MO-induced abrogation of Prox1 function, we performed a rescue experiment by coinjecting the embryos with 4 ng/embryo of *prox1 *MO and 400 pg/embryo of *prox1 *mRNA (Fig. 2D,H). 80% of the embryos at 36 hpf (n = 120) rescued the normal phenotype and displayed a proper number of hypothalamic TH-positive neurons (Fig. 2D,H). On the other hand, overexpression of *prox1 *alone does not lead to supernumerary CA neurons in the ventral diencephalon, nor determines ectopic CA neuron formation, confirming that *prox1 *functions are required for the proper development of the TH phenotype in a subpopulation of hypothalamic neurons, but are not sufficient to determine the appearance of supernumerary or ectopic TH-expressing cells. Interestingly, *prox1 *overexpression induces a slight increase of *th *mRNA levels, as detectable by means of quantitative real time RT-PCR only (data not shown); further investigations are necessary to elucidate this aspect that might reflect the ability of *prox1 *to directly modulate *th *expression in those few cells where the two genes are coexpressed.

In order to address whether *prox1 *is involved in neurogenesis processes, we analyzed the expression pattern of the proneural gene *ngn1 *[34]. *ngn1 *expression domains resulted unaffected in *prox1 *MO injected embryos, allowing us to conclude that loss of CA neurons in the hypothalamus of *prox1 *MO injected embryos is not caused by alteration in neurogenesis (see Additional file 5). Moreover, in order to address this issue, we analyzed the development of other neurotransmitter-producing neurons. Specifically, the neighboring serotonergic neurons appeared only slightly affected by Prox1 ablation in comparison to the most relevant effects we observed in the CA population (see Additional file 6). However, our description of *prox1 *effects on CA neuron development cannot rule out its potential involvement in the differentiation or fate determination of other neuronal types.

### prox1 functions are required for proper otp1 and TH phenotypes in the hypothalamic area

**Figure 3:**
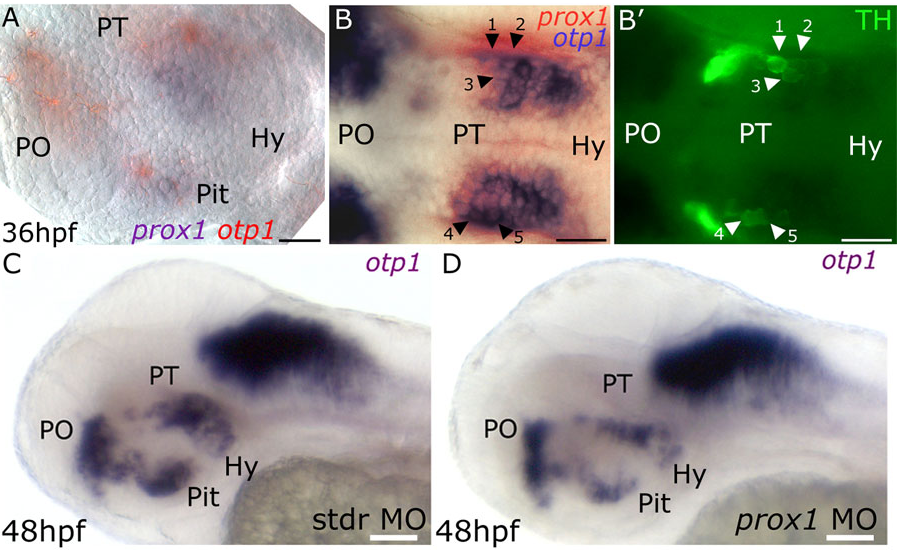
***prox1 *functions are required for proper *otp1 *and TH phenotypes in the hypothalamic area. **Anterior is left, dorsal is up except for *(B,B')*, dorsal view. In *(A) *eyes have been removed. *(A) prox1/otp1 *double WISH. Double staining of *prox1 *(blue) and *otp1 *(red) mRNAs at 36 hpf stage reveals that *otp1 *and *prox1 *colocalize in the hypothalamus. *(B,B') *36 hpf *prox1/otp1 *double WISH combined with TH immunohistochemistry. *(B) *A group of *prox1*/*otp1*-positive cells in the hypothalamus are also positive *(B') *for TH; for better orientation, some of these cells have been numbered (1–5) and indicated by black *(B) *and white *(B') *arrowheads, respectively. *otp1 *hypothalamic expression in *(C) *standard control morpholino and *(D) prox1 *MO injected embryos at 48 hpf. The following abbreviations are used: posterior tuberculum (PT), preoptic area (PO), standard control morpholino oligonucleotide (stdr MO). The following abbreviations are used: posterior tuberculum (PT), preoptic area (PO), pituitary (Pit), hypothalamus (Hy). Scale bars indicate 20 μm *(A,B,B') *or 30 μm *(C,D)*.

To further investigate the roles of *prox1 *in the hypothalamic CA neurons development, we showed that *prox1 *is also able to influence the *otp1 (otpb) *phenotype in hypothalamic neurons. As previously reported by our group [8], zebrafish *otp1 *contributes to the specification and differentiation of DA diencephalic neurons in the PT and the hypothalamus. In search of possible relationships between *prox1 *and *otp1 *in determining the TH phenotype, we performed a double WISH that evidenced the coexpression of the two genes in the hypothalamus (Fig. 3A,B). We also demonstrated that some of these *prox1/otp1*-positive cells are also positive for TH (Fig. 3B,B'), supporting the hypothesis that the coexpression of the two genes influences the development of the final TH phenotype. Injection of the embryos with *otp1 *MO or *otp1 *mRNA does not result in significant changes in *prox1 *expression pattern at 24 and 48 hpf (data not shown). On the other hand, a MO-mediated reduction of Prox1 levels determines significant modifications in *otp1 *expression pattern in the hypothalamic area, while *otp1 *expression in the rhombomeres resulted unperturbed. Specifically, the injection of *prox1 *MO causes the decrease of *otp1*-positive neurons in the hypothalamus at 48 hpf in comparison with embryos injected with the stdr MO (Fig. 3C,D). On the contrary, *prox1 *overexpression does not modify *otp1 *hypothalamic domains, nor determines *otp1 *ectopic expression (data not shown). Our findings indicate that *prox1 *is necessary, but not sufficient, to the proper *otp1 *phenotype in the hypothalamic area. Interestingly, *otp1 *appearance in the hypothalamus [8] precedes the onset of *prox1 *expression in this area, suggesting that *prox1 *is not involved in the activation of *otp1 *transcription. Rather, *prox1 *activity might be vital to control the switch of the *otp1*-positive cells towards their final TH fate. The hypothesis is supported by the evidence that *prox1 *MO and *otp1 *synthetic mRNA coinjection did not restore the normal TH phenotype (see Additional file 7). Thus, lack of Prox1 might impede further neuronal differentiation, causing apoptosis or cell misspecification, with consequent loss of *otp1 *and *th *expression. In order to discriminate between these two possibilities, we performed a TUNEL assay on *prox1 *MO injected embryos at 24 hpf (see Additional file 8) and at 36 hpf (data not shown). The level of apoptosis is not increased by Prox1 knock-down, suggesting that the lower number of *otp1*-positive hypothalamic neurons might be determined by misspecification events rather than apoptosis. Interestingly, *otp1 *is expressed in those cells already switched towards a more differentiated state, such as early postmitotic DA precursors, as well as newly specified and mature DA cells [8,11]. Thus, according to the wealth of literature data pinpointing *prox1 *as a key player in the passage from proliferation to differentiation [20,21,35], *prox1 *in the hypothalamus might drive the cells towards differentiative processes, leading to the terminal TH phenotype of those precursors committed to a CA phenotype by the expression of *otp1*.

### Overexpression of prox1 and otp1 together leads to supernumerary CA neurons in the ventral diencephalon and TH positive cells on the yolk surface

**Figure 4:**
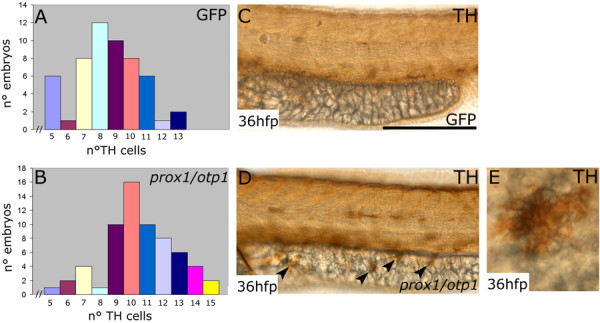
**Synergistic *prox1/otp1 *overexpression induces the appearance of hypothalamic supernumerary TH-positive neurons and ectopic TH-positive cells on the yolk surface ectoderm**. Lateral view, anterior is left and dorsal is up. *(A,B) *Distribution of TH positive cells in control and overexpressed *prox1*/*otp1 *embryos at 36 hpf. *(A) *The most numerous class in the group of GFP mRNA injected control embryos presented 8 CA hypothalamic neurons, and only 3 embryos presented more than 11 TH hypothalamic positive cells (n = 54). *(B) *The most numerous class in the group of the overexpressed *prox1*/*otp1 *embryos (n = 64) presented 10 CA neurons, and 20 embryos showed more than 11 TH hypothalamic positive cells. *(C,D) *Immunostaining with TH antibody shows ectopic TH positive cells on the yolk surface ectoderm of *prox1/otp1 *double injected embryos (arrowheads), while these cells are not present on the yolk of control embryos. *(E) *Ectopic TH positive cell on the yolk surface ectoderm. Scale bars indicate 50 μm.

To verify whether *prox1 *and *otp1 *together have an impact on the CA phenotype in the hypothalamus, we coinjected their specific mRNAs at a concentration of 450 and 300 pg/embryo, respectively, and stained the embryos for TH at 36 hpf. As reported above, the injection of *prox1 *mRNA alone did not increase the number of hypothalamic CA neurons, nor did single 300 pg/embryo *otp1 *mRNA injection (data not shown). Comparable results were obtained injecting 450 pg/embryo of the GFP mRNA as control (Fig. 4). The most numerous class in the group of control embryos presented 8 CA hypothalamic neurons, and only 3 embryos presented more than 11 TH hypothalamic positive cells. On the other hand, the most numerous class in the group of the overexpressed *prox1*/*otp1 *embryos (n = 64) presented 10 CA neurons, and 20 embryos showed more than 11 TH hypothalamic positive cells (Fig. 4A,B), pointing out the synergistic effect of the *prox1/otp1 *coexpression on the CA phenotype establishment. Remarkably, we also evidenced that *prox1*/*otp1 *coinjection induces TH positive cells on the yolk surface (Fig. 4D,E), while these ectopic cells were never observed in control (GFP) or single (*prox1 *or *otp1*) injected embryos (Fig. 4C). Noteworthy, in the CNS, the overproduction of TH positive cells induced by coinjection was detected in the hypothalamus, suggesting that *prox1 *and *otp1 *genes require additional factors, present in the ventral diencephalon, to induce the TH phenotype. Thus, we demonstrated that the coexpression of *prox1 *and *otp1 *determines a higher number of CA neurons in the hypothalamus and induces ectopic TH positive cells in non-neuroectodermal regions.

## Conclusion

In conclusion, we highlight for the first time the role of *prox1 *in the proper development of the CA neurons in the ventral diencephalon. Moreover, we provide evidence of regulatory links between *prox1 *and *otp1 *genes in defining the terminal TH phenotype in the hypothalamus. The identification of *prox1 *as a key component in the differentiation of hypothalamic CA neurons will help in clarifying the developmental bases of several human behavioral aspects as well as pathologies such as addictions and Parkinson's disease.

## Methods

### Animals

Breeding wild type fish of the AB strain were maintained at 28°C on a 14 h light/10 h dark cycle. Embryos were collected by natural spawning, staged according to Kimmel and colleagues [36], and raised at 28°C in fish water (Instant Ocean, 0,1% Methylene Blue) in Petri dishes. We express the embryonic ages in somites (s), hours post fertilization (hpf) and days post fertilization (dpf).

### RT-PCR

Total RNA from 17 samples (an average of 30 embryos per sample) corresponding to 10 different developmental stage embryos (1–2 cells, 30% epiboly, 50% epiboly, 80% epiboly, tailbud, 8 s, 15 s, 24 hpf, 72 hpf, and 5 dpf) and 7 adult organs (testis, ovary, gills, gut, eyes, brain, and liver) was extracted with the TOTALLY RNA isolation kit (Ambion), treated with RQ1 RNase-Free DNase (Promega) and oligo(dT)-reverse transcribed using SuperScript II RT (Invitrogen), according to manufacturers' instructions. The following primers were used for PCR reactions: *prox1_*sense 5'-ACCTCAGCCACCATCGTTCCATC-3' and *prox1_*antisense 5'-CACTATTCATGCAGAAGCTCCTGC-3'. PCR products were loaded and resolved onto 2% agarose gels.

### In situ hybridization and immunohistochemistry

Whole mount in situ hybridization (WISH), was carried out as described [23] on embryos fixed for 2 h in 4% paraformaldehyde/phosphate buffered saline, then rinsed with PBS-Tween, dehydrated in 100% methanol and stored at -20°C until processed for WISH [37]. Antisense riboprobes were previously *in vitro *labelled with modified nucleotides (*i.e*. digoxigenin, fluorescin, Roche). For histological sections, stained embryos were re-fixed in 4% PFA, dehydrated and stored in methanol, wax embedded and sectioned (5 μm). For immunohistochemistry, embryos were exposed to rabbit anti-Tyrosine Hydroxilase (anti-TH) (Chemicon), or rabbit anti-Serotonin (anti-5HT) (Chemicon), then treated with biotinylated or fluorescent secondary antibody (Vector Laboratories).

### Quantitative real time RT-PCR

Reverse transcriptions (RTs) were performed using 2 μg of DNase treated (DNA-*free*™, Ambion Inc) total RNA in presence of random hexamers (Invitrogen™) and SuperScript II reverse transcriptase (Invitrogen™). Real-time PCRs were carried out in a total volume of 15 μl containing 1× iQ SYBR Green Super Mix (BioRad), using 1 μl of the RT reaction. PCRs were performed using the BioRad iCycler iQ Real Time Detection System (BioRad Laboratories). For normalization purposes, 18S ribosomal RNA level was tested in parallel with the gene of interest. The following primers were used:

*th1_*sense 5'-ATGCCATCATCTTGTCACCA-3'

*th1_*antisense 5'-GGCAATGTCTCCGATCATCT-3';

18S_sense 5'-ACCTCACTAAACCATCCAATC-3'

18S_antisense 5'-AGGAATTCCCAGTAAGCGCA-3'.

### TUNEL staining

For TUNEL assay, 24 and 36 hpf embryos were fixed with 4% PFA for 2 h at room temperature. Embryos were permeabilized with methanol at -20°C and washed twice with PBC (0.001% Triton ×-100, 0.1% sodium citrate in PBS) for 10 minutes. Labeling for apoptotic cells was performed using In situ Cell Death Detection Kit (Roche). The embryos were incubated at 37°C for 1 h, washed and mounted for fluorescent microscopic imaging.

### Injections

Injections were carried out on 1- to 2-cell stage embryos; the dye tracer rhodamine dextran was also coinjected. Synthetic capped *prox1 *or *otp1 *mRNA were injected repeatedly (n > 3) at concentrations of 450 pg and 300 pg per embryo, respectively. Double mRNA injection was performed with 450 pg *prox1 *mRNA and 300 pg *otp1 *mRNA per embryo; 450 pg of synthetic capped GFP mRNA was injected as control. To repress *prox1 *mRNA translation, an ATG-targeting morpholino was synthesized (Gene Tools, LLC): 5'-ATGTGCTGTCATGGTCAGGCATCAC-3' [32,33]. *prox1 *MO was used at the concentration of 1 pmole in 1× Danieau buffer (pH 7,6) as previously reported [38]. To repress *otp1 *mRNA translation, an ATG-targeting morpholino was designed (Gene Tools, LLC): 5'-CCAAGAGGTCGGCATGAGAGAGCAT-3' [8]. *otp1 *MO was used at the concentration of 0,7 pmole. As control we injected a standard control morpholino oligonucleotide (stdr MO). Double *prox1 *MO/*otp1 *mRNA was performed with 1 pmole of MO and 300 pg of synthetic mRNA per embryo.

## Authors' contributions

AP, FC, and LDG designed the study. AP and LDG carried out functional studies and drafted the manuscript. AP, EB, GG, LDG, and SC performed WISH and Real Time RT-PCR expression analyses. All authors read and approved the final manuscript.

## Supplementary Material

Additional file 1 *prox1 *expression in the adaxial cells. Transverse section through the caudal trunk of the embryo. *(A*) 20 s embryo. *prox1 *signal is present only in the adaxial cells. This is approximately the time that these cells elongate in the anteroposterior dimension. *(B) *24 hpf embryo. The adaxial cells expressing *prox1 *are now lateral. Dorsal is always up. Scale bar indicates 50 μm. Click here for file

Additional file 2 *prox1 *expression in cranial ganglia. 48 hpf embryo lateral *(A) *and dorsal *(B) *view, respectively. *prox1 *mRNA is expressed in presumptive cranial motor and sensory neurons (boxed regions). Anterior is always to the left. Scale bar indicates 50 μm. Click here for file

Additional file 3 *prox1 *is required during the development of hypothalamic CA neurons. Lateral view in all panels. Anterior is to the left, and dorsal is up. Microinjection of *prox1 *MO lowers the number of hypothalamic CA neurons. At 24 and 48 hpf the TH-labelled cells in the hypothalamic/PT area are reduced in number in the *prox1 *MO injected embryos *(B,D) *when compared to the control embryos injected with stdr MO *(A,C)*. The following abbreviation is used: posterior tuberculum (PT). Scale bar indicates 10 μm. Click here for file

Additional file 4 *shh *expression pattern in the CNS of stdr MO and *prox1 *MO injected embryos. Anterior is left and dorsal is up in all panels. *(A) *24 hpf embryo injected with standard control morpholino oligonucleotide. *(B) *24 hpf embryo injected with *prox1 *MO. The overall brain patterning of the ventral diencephalon is not affected in *prox1 *MO injected embryos, as suggested by the normal expression of *shh *that we used as marker of proper differentiation of the ventral diencephalon. The following abbreviations are used: standard control morpholino oligonucleotide (stdr MO). Scale bars indicate 50 μm. Click here for file

Additional file 5 *ngn1 *expression pattern in the CNS of stdr MO and *prox1 *MO injected embryos. Anterior is left and dorsal is up in all panels. *(A) *24 hpf embryo injected with standard control morpholino oligonucleotide. *(B) *24 hpf embryo injected with *prox1 *MO. Neurogenesis is not disturbed in *prox1 *MO injected embryos as shown by normal *ngn1 *hypothalamic expression. The following abbreviation is used: standard control morpholino oligonucleotide (stdr MO). Scale bars indicate 50 μm. Click here for file

Additional file 6 5HT expression in *prox1 *MO injected embryos. Ventral view in all panels. Anterior is up. Anti-5HT antibody labels the hypothalamic serotonergic neurons at 48 hpf. *(B) *microinjection of *prox1 *MO does not significantly lower the number of 5HT-labelled neurons in the hypothalamus in comparison to the 48 hpf embryos injected with the same concentration of standard control morpholino oligonucleotide *(A)*. Scale bar indicates 10 μm. Click here for file

Additional file 7 TH expression in *prox1 *MO/*otp1 *mRNA coinjected embryos. Ventral view in all panels. Anterior is up. Anti-TH antibody labels the PT and hypothalamic CA neurons at 36 hpf (boxed regions). *(B) *microinjection of *prox1 *MO lowers the number of TH-labelled CA neurons in the hypothalamus in comparison to the 36 hpf embryos injected with the same concentration of standard control morpholino oligonucleotide *(A)*. *(C) *coinjection of *prox1 *MO and *otp1 *synthetic mRNA did not restore the normal TH phenotype. Scale bar indicates 20 μm. Click here for file

Additional file 8 *prox1 *MO injected embryos do not show increased apoptosis. Lateral view in all panels. Anterior is to the left, and dorsal is up. Apoptosis in 24 hpf embryos has been evaluated by means of TUNEL assay. *(B) prox1 *MO injected embryos do not show increases in apoptosis when compared to the control embryos injected with stdr MO *(A)*. The white drawing indicates the profile of the embryos. The following abbreviations are used: standard control morpholino oligonucleotide (stdr MO), eye (E), hypothalamus (Hy), yolk (y). Scale bar indicates 50 μm. Click here for file
