# Supplementary figures and images for: Crucial role of zebrafish prox1 in hypothalamic catecholaminergic neurons development
Source: BMC Dev Biol. 2008 Mar 10;8:27. doi: 10.1186/1471-213X-8-27 (PMC2288594; doi:10.1186/1471-213X-8-27)

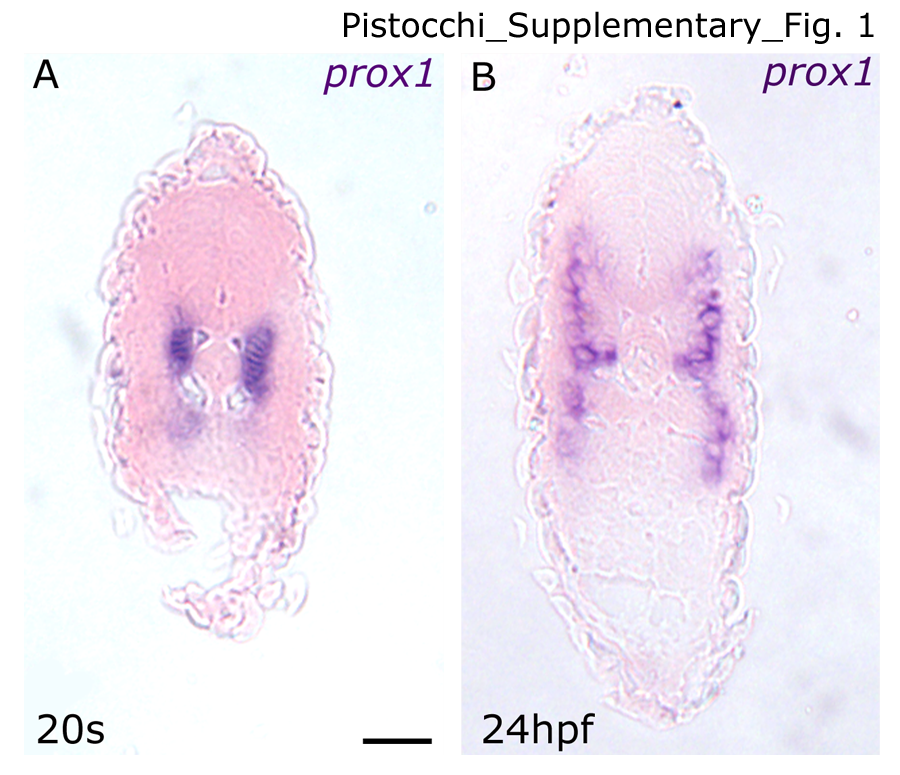

Supplement: Additional file 1 — prox1 expression in the adaxial cells. Transverse section through the caudal trunk of the embryo. (A) 20 s embryo. prox1 signal is present only in the adaxial cells. This is approximately the time that these cells elongate in the anteroposterior dimension. (B) 24 hpf embryo. The adaxial cells expressing prox1 are now lateral. Dorsal is always up. Scale bar indicates 50 μm. [file 1471-213X-8-27-S1.tiff]

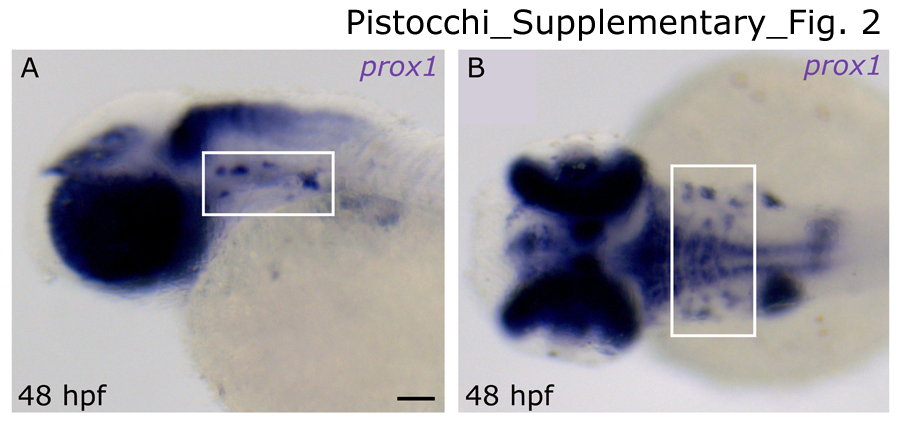

Supplement: Additional file 2 — prox1 expression in cranial ganglia. 48 hpf embryo lateral (A) and dorsal (B) view, respectively. prox1 mRNA is expressed in presumptive cranial motor and sensory neurons (boxed regions). Anterior is always to the left. Scale bar indicates 50 μm. [file 1471-213X-8-27-S2.tiff]

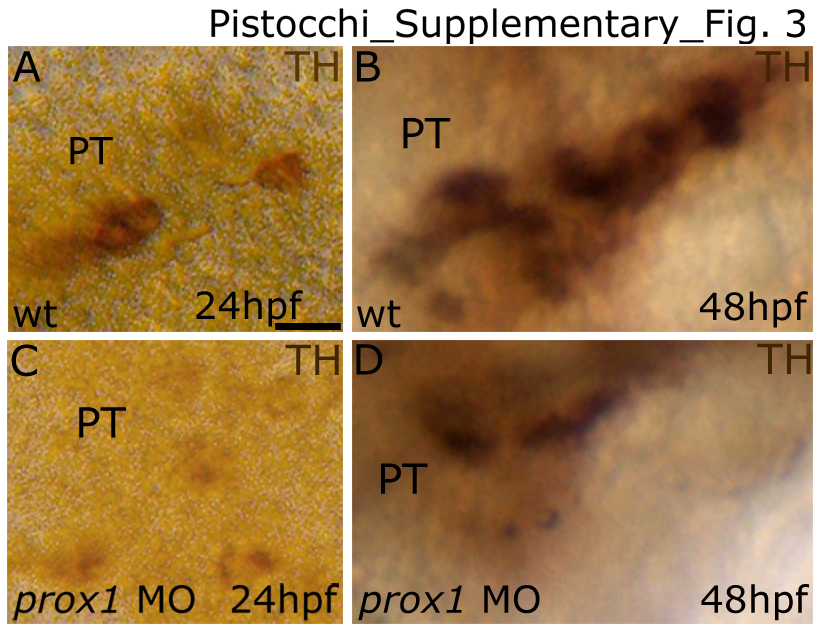

Supplement: Additional file 3 — prox1 is required during the development of hypothalamic CA neurons. Lateral view in all panels. Anterior is to the left, and dorsal is up. Microinjection of prox1 MO lowers the number of hypothalamic CA neurons. At 24 and 48 hpf the TH-labelled cells in the hypothalamic/PT area are reduced in number in the prox1 MO injected embryos (B,D) when compared to the control embryos injected with stdr MO (A,C). The following abbreviation is used: posterior tuberculum (PT). Scale bar indicates 10 μm. [file 1471-213X-8-27-S3.tiff]

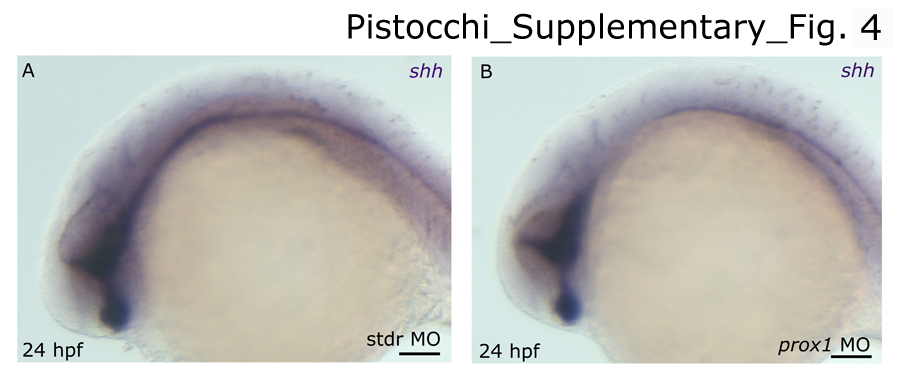

Supplement: Additional file 4 — shh expression pattern in the CNS of stdr MO and prox1 MO injected embryos. Anterior is left and dorsal is up in all panels. (A) 24 hpf embryo injected with standard control morpholino oligonucleotide. (B) 24 hpf embryo injected with prox1 MO. The overall brain patterning of the ventral diencephalon is not affected in prox1 MO injected embryos, as suggested by the normal expression of shh that we used as marker of proper differentiation of the ventral diencephalon. The following abbreviations are used: standard control morpholino oligonucleotide (stdr MO). Scale bars indicate 50 μm. [file 1471-213X-8-27-S4.tiff]

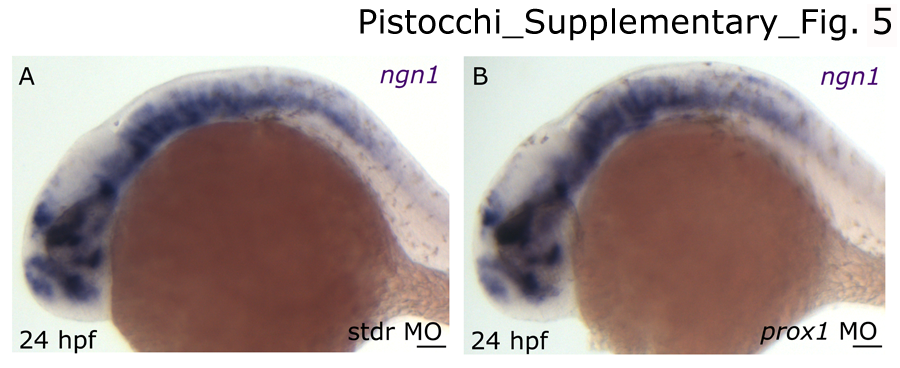

Supplement: Additional file 5 — ngn1 expression pattern in the CNS of stdr MO and prox1 MO injected embryos. Anterior is left and dorsal is up in all panels. (A) 24 hpf embryo injected with standard control morpholino oligonucleotide. (B) 24 hpf embryo injected with prox1 MO. Neurogenesis is not disturbed in prox1 MO injected embryos as shown by normal ngn1 hypothalamic expression. The following abbreviation is used: standard control morpholino oligonucleotide (stdr MO). Scale bars indicate 50 μm. [file 1471-213X-8-27-S5.tiff]

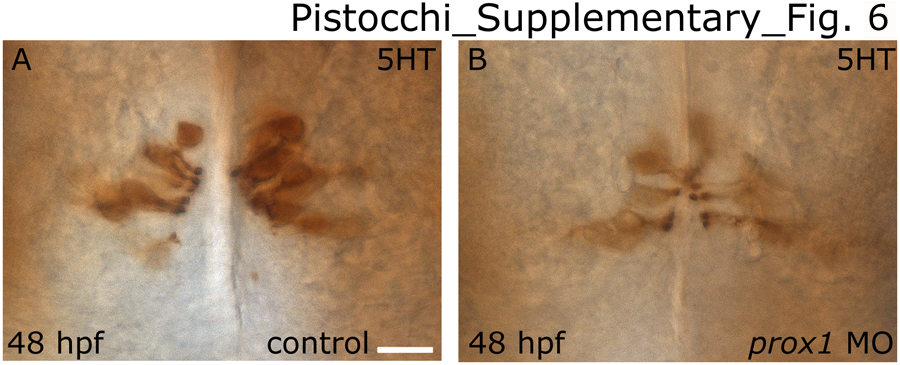

Supplement: Additional file 6 — 5HT expression in prox1 MO injected embryos. Ventral view in all panels. Anterior is up. Anti-5HT antibody labels the hypothalamic serotonergic neurons at 48 hpf. (B) microinjection of prox1 MO does not significantly lower the number of 5HT-labelled neurons in the hypothalamus in comparison to the 48 hpf embryos injected with the same concentration of standard control morpholino oligonucleotide (A). Scale bar indicates 10 μm. [file 1471-213X-8-27-S6.tiff]

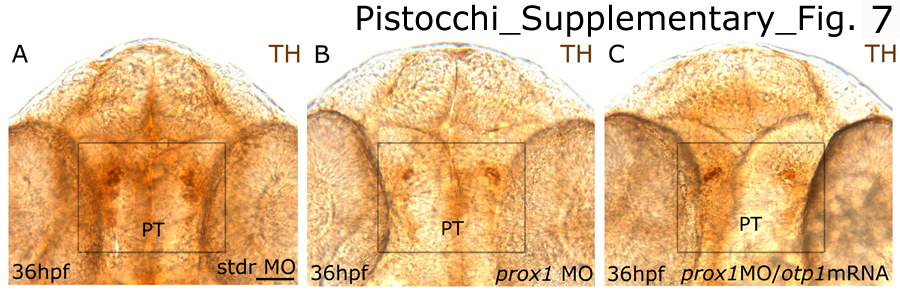

Supplement: Additional file 7 — TH expression in prox1 MO/otp1 mRNA coinjected embryos. Ventral view in all panels. Anterior is up. Anti-TH antibody labels the PT and hypothalamic CA neurons at 36 hpf (boxed regions). (B) microinjection of prox1 MO lowers the number of TH-labelled CA neurons in the hypothalamus in comparison to the 36 hpf embryos injected with the same concentration of standard control morpholino oligonucleotide (A). (C) coinjection of prox1 MO and otp1 synthetic mRNA did not restore the normal TH phenotype. Scale bar indicates 20 μm. [file 1471-213X-8-27-S7.tiff]

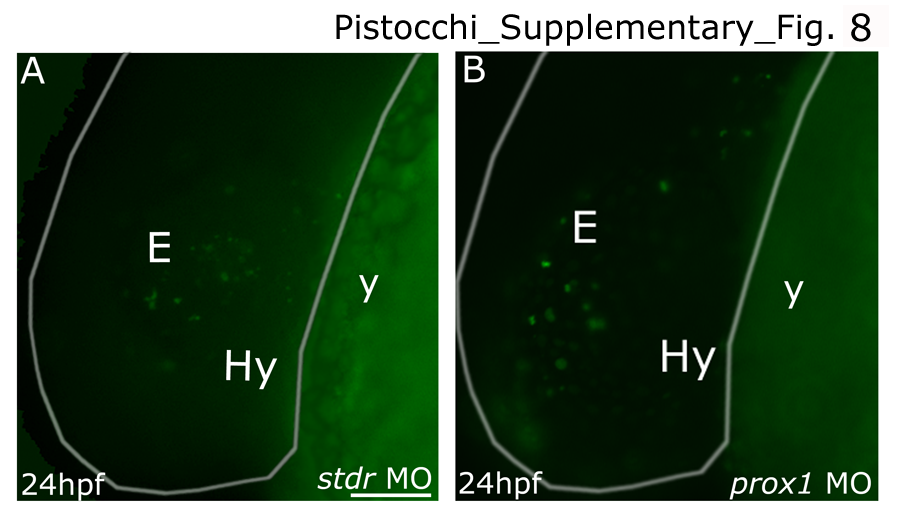

Supplement: Additional file 8 — prox1 MO injected embryos do not show increased apoptosis. Lateral view in all panels. Anterior is to the left, and dorsal is up. Apoptosis in 24 hpf embryos has been evaluated by means of TUNEL assay. (B) prox1 MO injected embryos do not show increases in apoptosis when compared to the control embryos injected with stdr MO (A). The white drawing indicates the profile of the embryos. The following abbreviations are used: standard control morpholino oligonucleotide (stdr MO), eye (E), hypothalamus (Hy), yolk (y). Scale bar indicates 50 μm. [file 1471-213X-8-27-S8.tiff]
